# Inflammatory and Metabolic Biomarker Assessment in a Randomized Presurgical Trial of Curcumin and Anthocyanin Supplements in Patients with Colorectal Adenomas

**DOI:** 10.3390/nu15183894

**Published:** 2023-09-07

**Authors:** Debora Macis, Irene Maria Briata, Oriana D’Ecclesiis, Harriet Johansson, Valentina Aristarco, Tania Buttiron Webber, Massimo Oppezzi, Sara Gandini, Bernardo Bonanni, Andrea DeCensi

**Affiliations:** 1 Division of Cancer Prevention and Genetics, IEO, European Institute of Oncology IRCCS, 20141 Milan, Italy; debora.macis@ieo.it (D.M.); harriet.johansson@ieo.it (H.J.); valentina.aristarco@ieo.it (V.A.); bernardo.bonanni@ieo.it (B.B.); 2 Division of Medical Oncology, E.O. Galliera Hospital, 16128 Genoa, Italy; irene.maria.briata@galliera.it (I.M.B.); tania.buttiron@galliera.it (T.B.W.); 3 Department of Experimental Oncology, IEO, European Institute of Oncology IRCCS, 20139 Milan, Italy; oriana.decclesiis@ieo.it (O.D.); sara.gandini@ieo.it (S.G.); 4 Division of Gastroenterology, E.O. Galliera Hospital, 16128 Genoa, Italy; massimo.oppezzi@galliera.it

**Keywords:** curcumin, anthocyanins, adenomatous polyps, colorectal neoplasms, interleukin-6, inflammation, primary prevention

## Abstract

Colorectal cancer prevention is crucial for public health, given its high mortality rates, particularly in young adults. The early detection and treatment of precancerous lesions is key to preventing carcinogenesis progression. Natural compounds like curcumin and anthocyanins show promise in impeding adenomatous polyp progression in preclinical models. We conducted a randomized, double-blind, placebo-controlled, phase II presurgical trial in 35 patients with adenomatous polyps to explore the biological effects of curcumin and anthocyanins on circulating biomarkers of inflammation and metabolism. No significant difference in biomarker changes by treatment arm was observed. However, the network analysis before treatment revealed inverse correlations between adiponectin and BMI and glycemia, as well as direct links between inflammatory biomarkers and leptin and BMI. In addition, a considerable inverse relationship between adiponectin and grade of dysplasia was detected after treatment (corr = −0.45). Finally, a significant increase in IL-6 at the end of treatment in subjects with high-grade dysplasia was also observed (*p* = 0.02). The combined treatment of anthocyanins and curcumin did not result in the direct modulation of circulating biomarkers of inflammation and metabolism, but revealed a complex modulation of inflammatory and metabolic biomarkers of colon carcinogenesis.

## 1. Introduction

Colorectal cancer (CRC) prevention plays a pivotal role in safeguarding public health and reducing the burden of this deadly disease. CRC, the second leading cause of cancer-related deaths worldwide and the third most commonly diagnosed, often develops silently and progresses rapidly [1,2]. However, by emphasizing prevention, we can significantly lower the incidence and mortality rates associated with this cancer. A concerning trend in the future is the anticipated rise in colorectal cancer incidence among younger individuals, potentially attributed to lifestyle changes, genetic predisposition, and environmental factors, highlighting the need for increased awareness and early screening initiatives [2]. Regular screening tests, such as colonoscopies and stool tests, enable the early detection of precancerous lesions, allowing for timely intervention and improved treatment outcomes [3].

The adenoma–carcinoma sequence is a well-recognized mechanism for CRC development [4]. Adenomatous polyps have the potential to undergo malignant transformation over time. This transformation occurs when certain genetic mutations accumulate within the cells of the polyp, leading to uncontrolled cell growth and the potential for invasive cancer development. Identifying and removing adenomatous polyps during screening tests, such as colonoscopies, is crucial for preventing the progression of colorectal cancer and related deaths [5]. The early detection and removal of these precancerous lesions can significantly reduce the risk of developing advanced-stage CRC, underscoring the importance of regular screenings for early intervention and prevention.

Circulating biomarkers play a pivotal role in understanding the intricate mechanisms underlying colon cancer development and progression. Metabolic biomarkers, such as glycemia, insulin, and the homeostasis model assessment of insulin resistance (HOMA-IR) index, provide insights into the metabolic dysregulation that can fuel cancer growth [6]. Inflammatory markers like high-sensitivity C reactive protein (hs-CRP), interleukin-10 (IL-10), interleukin-6 (IL-6), and tumor necrosis factor-alpha (TNF-α) reflect the chronic inflammation associated with tumor initiation and progression [7]. These molecules often intertwine with cell growth and proliferation, as evidenced by insulin-like growth factor I (IGF-I) and insulin-like growth factor-binding protein 3 (IGFBP-3), which modulate cell signaling pathways [8]. Adipokines, including adiponectin and leptin, originating from adipose tissue, influence the tumor microenvironment and energy balance, potentially impacting cancer progression [9]. Additionally, 25-hydroxyvitamin D (25OHD) levels are emerging as a factor in colon cancer, with potential regulatory effects on various cellular processes [10].

The use of natural compounds for CRC prevention has gained significant attention in recent years due to their potential health benefits [11]. There is growing interest in exploring the potential preventive role of natural compounds in impeding the progression of adenomatous polyps to CRC. Several studies have highlighted the beneficial effects of certain natural compounds in suppressing the growth of adenomatous polyps, through the modulation of various molecular pathways. For instance, compounds like curcumin, found in turmeric, have demonstrated anti-inflammatory and antioxidant properties that may help inhibit the formation of polyps and reduce their progression to cancer [12]. Anthocyanins, the natural pigments found in various fruits and vegetables, have been shown to potentially play a beneficial role in preventing CRC through their antioxidant and anti-inflammatory properties, as well as their ability to inhibit the growth of cancer cells and induce apoptosis [13]. In a previous randomized trial involving patients with adenomatous polyps, the combined supplementation of curcumin and anthocyanins demonstrated promising results in modulating tissue biomarkers of inflammation and proliferation in colon adenomas [14]. Our study found that the combination of these natural compounds led to a potentially favorable effect on the markers associated with the development and progression of colorectal cancer. The anti-inflammatory and anti-proliferative properties of curcumin and anthocyanins appeared to work in combination, providing a potential avenue for reducing the risk of colorectal cancer in individuals with adenomatous polyps.

In the present study, we aim to further enhance our understanding of the biological implications of curcumin and anthocyanin in patients with adenomatous polyps investigating circulating biomarkers related to inflammation and metabolism, thus improving valuable biological insights into their potential effects.

## 2. Materials and Methods

### 2.1. Study Design and Participants

A randomized, double-blind, placebo-controlled, phase II presurgical trial in patients with adenomatous polyps of the colon was conducted (MiRACol trial). Participants received either anthocyanin plus curcumin or a placebo for 4–6 weeks before undergoing polypectomy. To be eligible for the trial, patients had to be between 18 and 85 years old, have one or more adenomatous polyp in the colorectal tract with a diameter of at least 1 cm, and have an ECOG Performance Status ≤ 1. Patients with hyperplastic polyps, flat serrated adenomas, and cancer diagnosis were excluded from the study, as were those who had taken experimental medications, bilberry-based dietary supplements, or curcumin in the 15 days leading up to enrollment.

The duration of the experimental treatment was suited to current standard clinical practice. Polyps larger than 1 cm in diameter are typically removed in a subsequent colonoscopy, following the availability of blood coagulation test results, as a safety precaution. Additionally, the 4-week interval between polyp detection and polypectomy falls within the common waiting period observed in numerous national health system facilities.

Participants were recruited from the Department of Gastroenterology at Galliera Hospital in Genoa, Italy, between March 2014 and December 2017. The study registered as NCT01948661, was conducted under the approval of the Liguria Region Ethical Committee and in accordance with the Declaration of Helsinki. All patients provided written informed consent before participating in the trial.

### 2.2. Study Procedures

During screening, subjects underwent colonoscopy with evaluation for the presence of adenomatous polyp ≥1 cm and collection of tissue biopsies of the polyp and perilesional normal tissue. Once the histological confirmation and eligibility criteria were verified, eligible patients were randomly assigned to either the active treatment group (active arm) or the control group receiving a placebo (control arm). Participants were instructed to take 1 tablet of 500 mg Mirtoselect^®^ or the corresponding placebo, as well as 1 tablet of 500 mg Meriva^®^ or the matching placebo, before breakfast and dinner every day, totaling 4 tablets daily for 4–6 weeks before polyp removals. Participants were instructed to avoid using curcumin as a spice and were asked to keep a diary to record their consumption of fruits and vegetables rich in anthocyanins. Fasting blood samples for biomarker analysis were drawn at baseline and final visit. Further details about study procedures and dietary supplements have been previously published [14].

### 2.3. Dietary Supplements

Indena SpA (Milan, Italy) generously contributed curcumin (Meriva^®^), anthocyanin (Mirtoselect^®^), and a matching placebo.

Meriva^®^ is a patented complex of curcumin with soy phosphatidylcholine with an overall content of curcumin of 20%. The choice of a daily dose of 1 g of curcumin (Meriva^®^) was based on previous studies that demonstrate that the complexation of curcumin with phosphatidylcholine can increase the bioavailability of the active ingredient [15,16]. In comparison to prior studies using non-complexed curcumin, it allows for lower doses [17]. This contributes to overcoming the limitations of orally tested curcumin preparations, such as instability at intestinal pH levels, low water solubility, poor oral bioavailability, and rapid conjugation and excretion. Furthermore, curcumin extract has been proven to be safe and well-tolerated. Oral intake in previous clinical studies has not been associated with significant side effects [18,19].

Mirtoselect^®^ is a standardized extract from bilberry (*Vaccinium myrtillus*) containing 36% of anthocyanins, including cyanidin-3-glucoside (C3G). The choice of a daily dose of 1 g of anthocyanin (Mirtoselect^®^) was based on a substantial body of prior research that ensures its safety even at much higher doses than 1 g. Specifically, with blueberry extract, results on effectiveness, pharmacokinetics and safety from various clinical studies are available, starting from a dose of 160 mg with 2 daily administrations, and ranging up to a daily dose of 10.8 g for 7 days of treatment [20,21,22,23,24].

### 2.4. Circulating Biomarkers Assessment

Fasting blood samples were obtained at baseline and the end of the study after 4–6 weeks of intervention. Blood samples were processed into serum and aliquots were frozen at −80 °C until biomarkers measurements.

We measured hs-CRP, glycemia, and insulin using the Abbott Alinity analyzer (Abbott Diagnostics, Abbott Park, IL, USA). We then calculated the HOMA-IR index as an indicator of metabolic status [25].

Total IGF-I, IGFBP-3, and 25OHD concentrations were assessed by chemiluminescent immunometric assay on the IDS-iSYS analyzer (Immunodiagnostic Systems Limited, East Boldon, UK).

Adiponectin, leptin, IL-10, IL-6, and TNF-α were measured on the automated platform ELLA (ProteinSimple, BioTechne, Minneapolis, MN, USA), as previously described [26].

Samples from the same participant were analyzed in the same batch. The laboratory staff were blinded to the arm assignment.

### 2.5. Statistical Analysis

The sample size calculation was based on data from our previous randomized trial on the same study population [27]. We have assumed a baseline median expression of β-catenin in adenomatous tissue to be 25% (with a standard deviation of 35) and a correlation between baseline and final β-catenin values of approximately 0.9. With these assumptions, using an ANCOVA model (therefore adjusting the analysis for baseline β-catenin levels), with 50 subjects per group we had a statistical power of 85% to observe an average difference of 10% between the two groups in β-catenin expression levels in adenomatous tissue. The sample size calculation had taken into account a 10% loss of subjects during follow-up and a two-tailed alpha error of 5%. The study was initially envisioned as a multicentric trial, but due to regulatory and logistical constraints, only one center was able to recruit. As a result, the overall number of randomized subjects was limited to 35 with consequent limitation in reaching the power to appreciate biomarker differences.

We presented median values and interquartile ranges (IQR) of serum biomarkers at baseline and the end of the study, in changes in time from baseline and % changes, by treatment arm. Biomarker values below the limit of detection (LLOD) were obtained by dividing the LLOD by two. Correlations among biomarkers are measured through Spearman correlation coefficients, which are “joined” in a graphical representation to create a network. Differences by arms were evaluated by Wilcoxon rank tests and through ANCOVA models adjusted for baseline values and significant confounders. The normal distribution of residuals from the full model was graphically checked. Due to the exploratory nature of the study, adjustment for multiple testing was not performed.

Two-tailed *p*-value < 0.05 was considered statistically significant. The statistical analyses were performed with R software, version 4.3.0.

## 3. Results

Between March 2014 and December 2017, 35 subjects were randomized in the MiRACol trial. The baseline characteristics of study participants were not different by treatment arm, as reported in Table 1 and our previous publication [14].

Table 2 shows the median and IQR of biomarkers at baseline and at the end of treatment in terms of the median change and the median % change with IQRs. We did not observe significant differences in biomarkers levels or their change by treatment arm.

**Figure 1:**
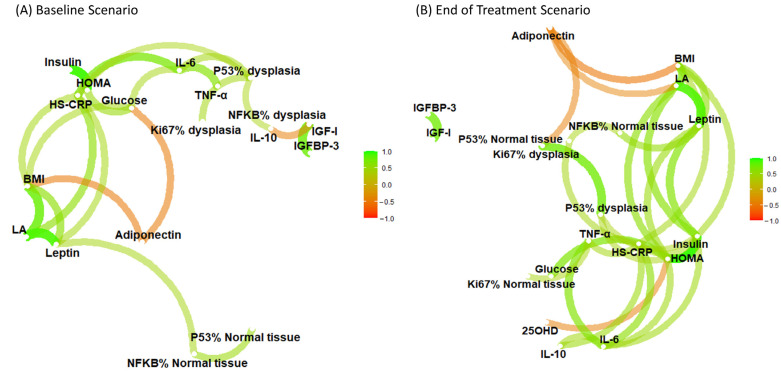
Network correlation analysis of biomarkers at baseline (**A**) and the end of treatment (**B**). In the network, nodes represent individual biomarkers, while Spearman correlation coefficients between biomarkers are depicted as edges (lines connecting nodes). The color spectrum of the edges ranges from green (direct correlation) to orange (inverse correlation). The thickness of the edges indicates the strength of the association. The position of a node reflects its centrality or rather the importance of a node within the network. Abbreviations: HOMA-index, Homeostasis Model Assessment Index; HS-CRP, high-sensitivity C reactive protein; IGF-1, insulin-like growth factor 1; IGFBP-3, insulin-like growth factor-binding protein 3; IL-6, interleukin-6; IL-10, interleukin-10; LA, Leptin Adiponectin Ratio; TNF-α, tumor necrosis factor-alpha; 25OHD, 25-hydroxyvitamin D; NFKB, nuclear factor kappa B.

Two correlation networks were drawn, based on Spearman correlations of biomarkers at baseline and the end of treatment (Figure 1). In the baseline network graph, we observed significant inverse correlations of adiponectin with BMI and glycemia, and direct correlations between inflammatory biomarkers (IL-6 and TNFα, IL-6, and hs-CRP), and between leptin and BMI (Figure 1A). The network analysis at the end of treatment showed similar correlations, except for a considerable inverse correlation between adiponectin and the post-treatment Ki67 levels in dysplasia tissue (Figure 1B).

The exploratory univariate analysis did not show any significant effect modification on biomarkers changes and treatment allocation arm given by socio-demographic variables. However, we observed a greater IL-10 increase in subjects with BMI ≤ 25 compared with BMI > 25 (Supplementary Figure S1). Furthermore, subjects with low anthocyanin food intake exhibited a greater reduction in leptin levels (Supplementary Figure S2).

**Figure 2:**
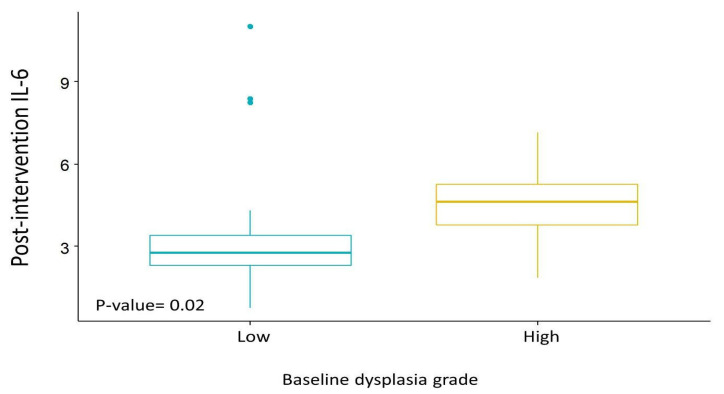
Mixed models analysis of post-intervention IL-6 by baseline dysplasia grade.

A mixed model analysis of biomarker changes revealed significantly higher IL-6 values at the end of the study in subjects with a high baseline dysplasia grade (*p* = 0.02 adjusted for baseline, BMI, and treatment arms; Figure 2).

We found that IGFBP-3 circulating levels at the end of the study were higher in subjects with a family history of CRC compared to subjects without a family history of CRC (*p* = 0.04 adjusted for baseline, age, and treatment arms, Supplementary Figure S3).

We observed a trend towards lower adiponectin levels in the active treatment arm compared to placebo (*p* = 0.09 adjusted for baseline adiponectin and BMI, Supplementary Figure S4).

## 4. Discussion

The findings from our study provide valuable insights into the complex relationships between biomarkers and treatment outcomes of curcumin and anthocyanin in patients with adenomatous polyps. Notably, no significant differences in biomarker levels or their changes were observed across the treatment arms, indicating that the interventions may not have directly influenced these specific biomarkers. However, our network analysis revealed compelling patterns, including direct correlations between adiponectin and BMI and glycemia, as well as direct links between inflammatory biomarkers and between leptin and BMI. These correlations potentially reflect disruptions in metabolic regulation that could contribute to adenomatous polyp development or progression. The direct link between leptin and BMI might indicate a complex interaction between the excess of body weight and signaling related to adenomatous polyp growth. These connections shed light on the complex interplay between metabolic and inflammatory pathways that could contribute to adenomatous polyp development or progression. These findings offer avenues for further research into preventive or therapeutic strategies targeting metabolic and inflammatory pathways contributing to the adenoma–carcinoma sequence in CRC development.

Moreover, we detected an inverse relationship between post-treatment adiponectin levels and Ki67 levels in dysplasia tissue, suggesting a potential role of this adipokine in dysplasia progression. Adiponectin is a hormone secreted by adipose tissue that plays a role in regulating glucose and lipid metabolism. It is also involved in various anti-inflammatory and anti-tumorigenic processes [28].

The finding of an inverse correlation between adiponectin and Ki67 levels in dysplasia tissue suggests a potential relationship between adiponectin levels and the severity of dysplasia. One possible explanation for this correlation is the role of adiponectin in inflammation. Adiponectin has been shown to have anti-inflammatory effects by suppressing the production of pro-inflammatory molecules and promoting the release of anti-inflammatory factors. Inflammation plays a crucial role in the development and progression of dysplasia, and lower levels of adiponectin could contribute to a pro-inflammatory environment that facilitates dysplasia [29]. Furthermore, adiponectin has been implicated in regulating cell proliferation and apoptosis, which are important processes in maintaining tissue homeostasis and preventing abnormal cell growth. Reduced adiponectin levels could disrupt these regulatory mechanisms and contribute to dysplasia progression [30]. It is important to note that this finding represents a correlation based on a small sample size, and further research is needed to confirm the relationship between adiponectin and dysplasia and clarify the underlying mechanisms involved.

Equally noteworthy was the observed increase in IL-6 at the end of treatment in subjects with high baseline dysplasia grade, suggesting a composite interplay between dysplasia severity and inflammatory processes. The expected outcome of treatment with the natural compounds curcumin and anthocyanins would be a reduction in dysplasia grade and a decrease in inflammation, given their anti-inflammatory properties. However, the observed increase in IL-6 could be counterintuitive, since IL-6 is associated with inflammation, and increased levels might suggest a pro-inflammatory response. The mechanism by which IL-6 could be involved in dysplasia progression is not fully understood. One possible explanation is that chronic inflammation sustained by granulocytes, plasma cells, lymphocytes, and macrophages results in high levels of pro-inflammatory cytokines. IL-6 in particular creates a microenvironment conducive to abnormal cell proliferation and promotes the survival of dysplastic cells, inhibiting apoptosis and possibly contributing to their progression toward cancerous changes [31].

The observation of a greater IL-10 increase in subjects with BMI < 25 compared to BMI > 25 is an interesting finding that suggests a potential relationship between body weight and the immune response. IL-10 is an anti-inflammatory cytokine that plays a crucial role in dampening the immune response and reducing inflammation [32]. The differential response based on BMI suggests a potential influence of body weight on the regulation of IL-10 levels. It is possible that subjects with a lower BMI might exhibit a more robust anti-inflammatory response, contributing to the higher IL-10 levels observed.

Surprisingly, our study did not yield evidence of curcumin inhibiting TNF-α, which could be primarily attributed to the limitations of a small sample size. Notably, recent research through a systematic review and meta-analysis of randomized controlled trials, indicated that with increasing doses of curcumin, there was a trend towards a greater reduction in TNF-α levels [33]. This suggests that the efficacy of curcumin on TNF-α might be dose-dependent, and the absence of this effect in our study might be due to the dosage employed. Furthermore, the meta-regression analysis highlighted that a higher baseline TNF-α level was associated with a more pronounced reduction after curcumin supplementation, suggesting a role of baseline biomarker levels in determining curcumin’s impact on TNF-α.

The finding that subjects with low anthocyanin food intake show a greater reduction in leptin levels suggests a potential relationship between anthocyanin dietary consumption and leptin regulation. Leptin is a hormone produced by adipose tissue that plays a central role in appetite regulation and energy balance. Higher leptin levels generally signal satiety and a reduced appetite, while lower levels may lead to increased hunger and food intake [34]. Anthocyanins are a group of plant pigments found in various fruits and vegetables. Numerous studies have highlighted the potential health benefits of anthocyanins, including their antioxidant, anti-inflammatory, and anti-obesity properties [35]. The observed greater reduction in leptin levels in individuals with low anthocyanin intake might imply that these compounds could influence leptin sensitivity or production, potentially affecting appetite and energy homeostasis through metabolic effects, regulation of adiposity, gut microbiota interactions, and inflammation modulation. However, it is essential to consider study limitations due to the small sample size and potential confounding factors. Individuals with low anthocyanin intake might have different dietary patterns or lifestyle factors that could influence leptin levels independently of anthocyanin intake. Additionally, the specific sources of anthocyanins consumed vary among the study participants, potentially affecting the outcomes.

It is interesting to note that subjects with a positive family history of colorectal cancer had greater circulating levels of IGFBP-3 at the end of the trial than subjects without a family history. IGFBP-3 is a multifunctional protein that regulates the activity of insulin-like growth factors (IGFs) and plays a crucial role in cell growth, differentiation, and apoptosis [36]. Recent research from nearly 400,000 people in the UK Biobank using complementing serologic and Mendelian randomization analyses showed that levels of IGFBP3 predicted based on genetic factors were associated with colorectal cancer risk [37]. The association of IGFBP-3 with a positive family history of colorectal cancer has not been a focus of significant research. We can speculate that because colorectal cancer is associated with chronic inflammation in the gut, inflammatory cytokines, and growth factors released in response to chronic inflammation may influence the expression and secretion of IGFBP-3.

The finding of a trend to lower adiponectin levels in subjects with colorectal adenomas taking curcumin and anthocyanins compared to placebo is an unexpected result. A comprehensive systematic review and meta-analysis of 60 randomized controlled trials found that curcumin/turmeric supplementation improved anthropometric indices of obesity and increased adiponectin levels in the general population [38]. Similarly, dietary anthocyanins significantly increase adiponectin levels, according to evidence from a systematic review and meta-analysis [39]. The reduction in adiponectin levels in the curcumin and anthocyanins treatment group raises questions about possible reasons. Responses to dietary interventions can vary among individuals due to genetic, metabolic, or lifestyle factors. The participants’ health status, dietary patterns, and other health-related factors could have influenced adiponectin levels. Moreover, the duration and dosage of the treatment might have influenced the outcomes. Longer or higher doses of curcumin and anthocyanins might have different effects on adiponectin levels. Finally, and perhaps most importantly, the small sample size of our study may have skewed the statistical results, making it impossible to draw more definitive conclusions.

The study has several limitations, including the small sample size and the short duration of intervention, which may have precluded the achievement of a significant modulation of circulating biomarkers of colon carcinogenesis related to inflammation and metabolism.

## 5. Conclusions

In conclusion, the combined treatment of curcumin and anthocyanins in patients with colorectal adenomas did not directly modulate circulating biomarkers. While the expected changes in biomarker levels were not observed, our study yielded intriguing results, shedding light on the complex interplay between metabolic and inflammatory pathways. The uncovered correlations between adiponectin, BMI, glycemia, inflammatory markers, and leptin provided valuable insights into the intricate regulatory mechanisms within the body. Additionally, the findings of an inverse relationship between adiponectin levels and dysplasia grade post treatment, along with an increase in IL-6 in subjects with high baseline dysplasia grade, warrant further investigation into the potential roles of these biomarkers in disease progression. Our research underscores the importance of understanding the multifaceted interactions between metabolic and inflammatory pathways in patients with colorectal adenomas and may guide the development of novel treatment strategies tailored to individual disease characteristics.

## Figures and Tables

**Table 1:** Baseline characteristics by treatment arm.

Parameter		Treatment		*p*-Value
		Active	Placebo	
*n* (%)		15 (51.7)	14 (48.3)	
Gender, *n* (%)	F	5 (33.3)	7 (50.0)	0.462
	M	10 (66.7)	7 (50.0)	
Age, mean ± SD		70.8 ± 9.8	67.9 ± 10.8	0.454
Baseline BMI, *n* (%)	<25	7 (46.7)	9 (64.3)	0.462
	≥25	8 (53.3)	5 (35.7)	
Family history of CRC, *n* (%)	No	10 (66.7)	7 (50.0)	0.462
	Yes	5 (33.3)	7 (50.0)	
Foods rich in anthocyanins, (servings per day)	<2	7 (46.7)	7 (50.0)	1.000
	≥2	7 (53.3)	7 (50.0)	
Histological type, *n* (%)	Tubular	10 (66.7)	10 (71.4)	1.000
	Villous	5 (33.3)	4 (28.6)	
Dysplasia grade, *n* (%)	Low-grade	12 (80.0)	9 (64.3)	0.427
	High-grade	3 (20.0)	5 (35.7)	

Data are presented as number (%), except those that were differently noted. Abbreviations: SD, standard deviation; F, female; M, male; BMI, body mass index; CRC, colorectal cancer.

**Table 2:** Circulating biomarker according to treatment arm and time point.

Serum Biomarkers Median (IQR)		Treatment		*p*-Value
		Active *n* = 16	Placebo *n* = 17	
HOMA-index	Pre	1.52 (1.21–2.45)	1.39 (1.03–1.66)	0.17
	Post	1.07 (0.77–1.31)	0.66 (0.48–0.97)	
	%change	−28%	−53%	
hs-CRP (mg/dL)	Pre	0.15 (0.10–0.25)	0.10 (0.10–0.10)	0.50
	Post	0.20 (0.10–0.60)	0.15(0.10–0.30)	
	%change	0%	25%	
L/A	Pre	0.70 (0.35–1.30)	0.80(0.40–2.30)	0.34
	Post	0.40 (0.20–0.70)	0.30(0.20–0.90)	
	%change	−51%	−63%	
Adiponectin (µg/mL)	Pre	12.80 (7.60–20.80)	12.60 (7.10–19.20)	0.94
	Post	9.70 (6.00–18.20)	13.20 (7.60–20.30)	
	%change	−12%	2%	
Leptin (ng/mL)	Pre	8.30 (3.75–11.00)	11.00 (5.00–22.0)	0.91
	Post	2.90 (1.30–7.20)	4.05 (2.00–12.60)	
	%change	−63%	−51%	
IGFBP-3 (ng/mL)	Pre	3269.15 (2860.80–3706.15)	3150.80 (2400.20–3546.20)	0.91
	Post	3227.50 (2678.70–3545.30)	2891.25 (2698.90–3731.90)	
	%change	−6%	−5%	
IGF-1 (ng/mL)	Pre	112.65 (99.65–146.15)	109.60 (79.90–124.50)	0.27
	Post	110.20 (79.30–137.90)	98.85 (73.70–140.70)	
	%change	−12%	−7%	
25OHD (ng/mL)	Pre	16.80 (7.55–22.05)	15.20 (8.70–19.20)	0.92
	Post	15.10 (9.20–22.90)	16.55 (11.00–26.60)	
	%change	0%	4%	
IL-10 (pg/mL)	Pre	3.15 (2.70–4.30)	3.20 (2.30–3.70)	0.29
	Post	4.10 (2.70–6.20)	3.20 (2.30–3.90)	
	%change	10%	21%	
IL-6 (pg/mL)	Pre	3.50 (2.85–5.00)	2.90 (1.90–3.60)	0.60
	Post	3.90 (2.50–6.30)	2.80 (2.00–3.50)	
	%change	14%	6%	
TNFα (pg/mL)	Pre	11.30 (9.60–14.50)	10.50 (9.90–12.50)	0.42
	Post	11.70 (8.80–14.20)	11.05 (9.60–11.70)	
	%change	−1%	−2%	

*p*-value of changes adj for baseline. Abbreviations: hs-CRP, high-sensitivity C reactive protein; HOMA-index, homeostasis model assessment index; IGF-1, insulin-like growth factor 1; IGFBP-3,iInsulin-like growth factor-binding protein 3; IL-6, interleukin 6; IL-10, interleukin 10; IQR, interquartile range; L/A, Leptin Adiponectin Ratio; TNFα, tumor necrosis factor alpha; 25OHD, 25-hydroxyvitamin D.

## Data Availability

Data may be made available for collaborative studies upon reasonable request to the corresponding author.

## Supplementary Materials

The following supporting information can be downloaded at: https://www.mdpi.com/article/10.3390/nu15183894/s1, Figure S1: Univariate analysis of IL-10 changes by BMI; Figure S2: Univariate analysis of leptin changes by dietary anthocyanin intake; Figure S3: Mixed models analysis of post-intervention IGFBP-3 by colorectal cancer family history; Figure S4: Mixed models analysis of adiponectin by treatment arm.
