# Supplementary material for: Inflammatory and Metabolic Biomarker Assessment in a Randomized Presurgical Trial of Curcumin and Anthocyanin Supplements in Patients with Colorectal Adenomas
Source: Nutrients. 2023 Sep 7;15(18):3894. doi: 10.3390/nu15183894 (PMC10537228; doi:10.3390/nu15183894)

Supplementary Materials

Supplementary Figure S1. Univariate analysis of IL-10 changes by BMI

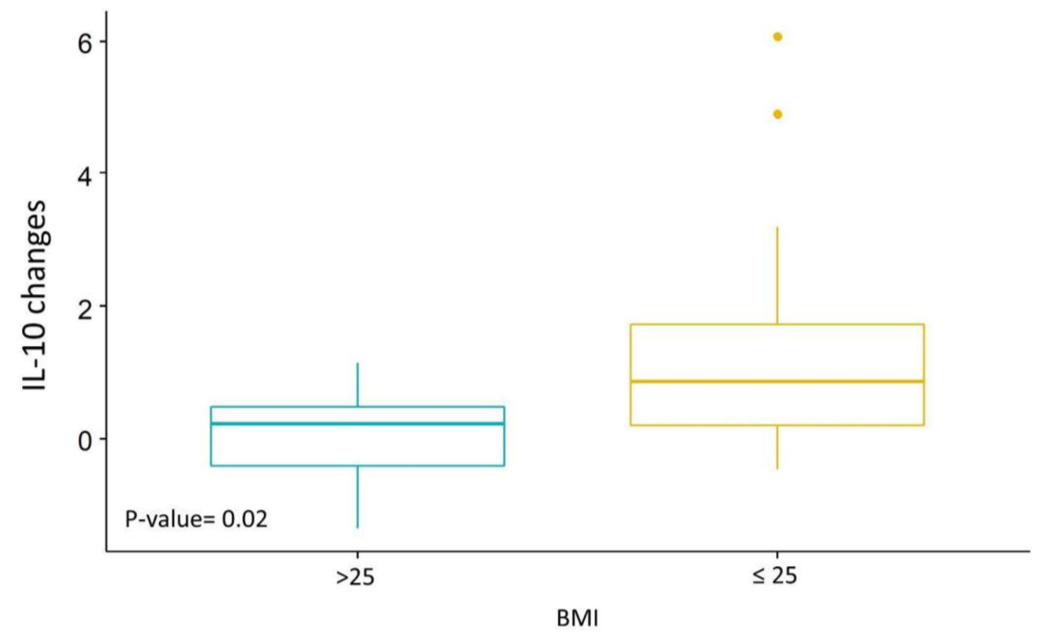

Supplementary Figure S2. Univariate analysis of leptin changes by dietary anthocyanin intake

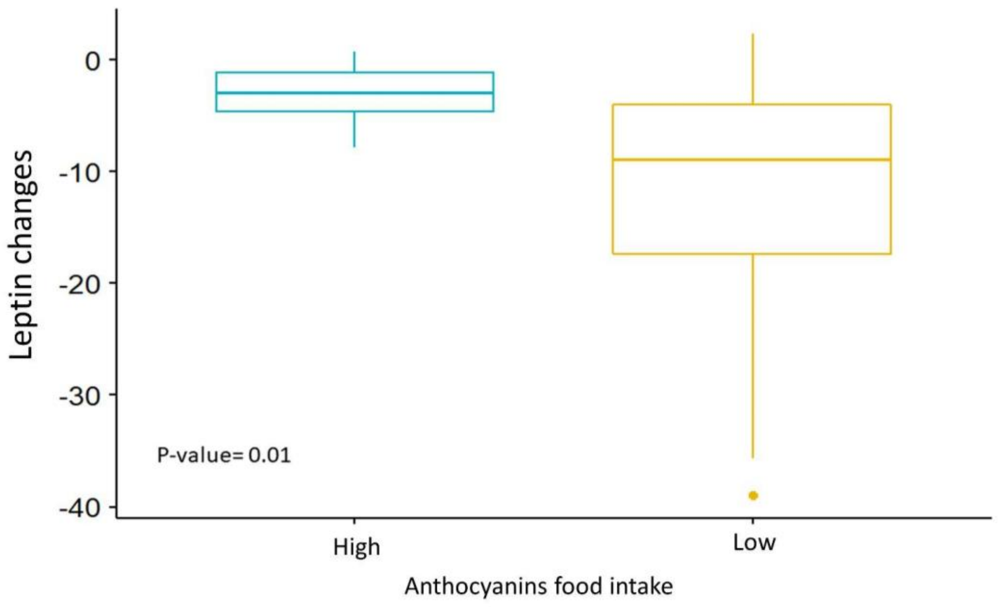

Supplementary Figure S3. Mixed models analysis of post-intervention IGFBP-3 by colorectal cancer family history

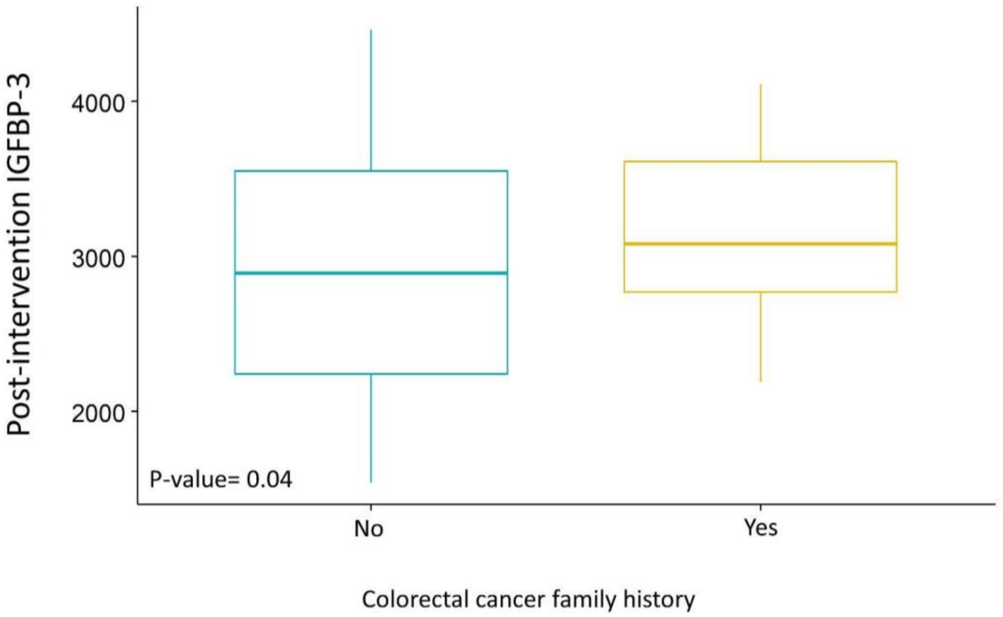

Supplementary Figure S4. Mixed models analysis of adiponectin by treatment arm

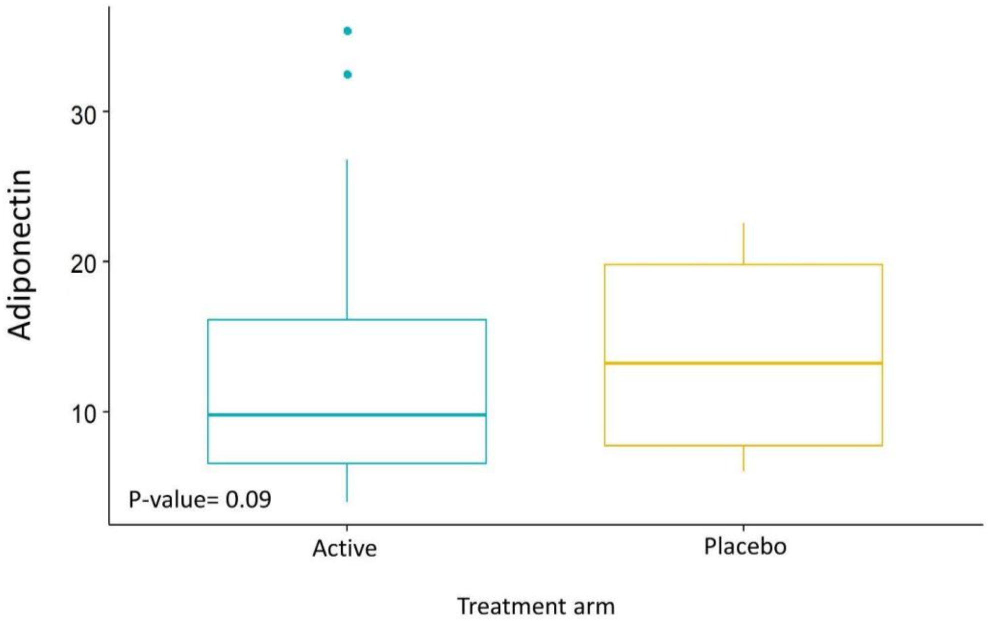

Supplement: Supplementary file 1 [file nutrients-15-03894-s001.zip › nutrients-2589714-supplementary.pdf]
